# BDWaste: A comprehensive image dataset of digestible and indigestible waste in Bangladesh

**DOI:** 10.1016/j.dib.2024.110153

**Published:** 2024-02-07

**Authors:** Wahidur Rahman, Mohona Akter, Nahida Sultana, Maisha Farjana, Arfan Uddin, Md. Bakhtiar Mazrur, Mohammad Motiur Rahman

**Affiliations:** a Department of Computer Science and Engineering, Uttara University, Dhaka, Bangladesh; b Department of Computer Science and Engineering, Mawlana Bhashani Science & Technology University, Tangail, Bangladesh

**Keywords:** Sustainable development, Machine learning, Waste classification, Deep learning

## Abstract

The “BDWaste” dataset contains two significant categories of waste, namely digestible and indigestible, in Bangladesh. Each category represents 10 distinct species of waste. The digestible categories are sugarcane husk, fish ash, potato peel, paper, mango peel, rice, shell of malta, lemon peel, banana peel, and egg shell. On the other hand, the indigestible species are polythene, cans, plastic, glass, wire, gloves, empty medicine packets, chip packets, bottles, and masks. The research uploaded the primarily collected dataset on Mendeley, and the dataset contains a total of 2497 raw images, of which 1234 were digestible and 1263 belonged to indigestible species. Each species is stored in a fixed file based on its name and categories. Also, each species contains an indoor (with a visible surface) and an outdoor (with a surface that can be seen generally) image. The dataset is free from any blurry, dark, noisy, or invisible images. The research also performed waste classification with pre-trained convolutional neural network models such as MobileNetV2 and InceptionV3. The research found the highest accuracy of 96.70% in the indigestible waste classification and 99.70% in the digestible waste classification. The researchers presume that this data can be used in the future in different types of research, such as sustainable development, sustainable environments, agricultural development, recycling processes, and other computer vision-based applications.

Specification Table

**Table:** 

Subject	Computer Sciences, Agricultural Sciences, Food and nutrition science
Specific subject area	Computer Vision classification of different types of Wastes.
Data format	Raw, Analyzed, and Filtered.
Type of data	Image.
Data collection	Selected waste found in the local area. The wastes are divided into two categories: digestible and indigestible. The “BDWaste” data set contains a total of 20 distinct types of waste. 1. Sugarcane husk, 2. Fish ash, 3. Potato peel, 4. Paper, 5. Lemon Peel, 6. Mango Peel, 7. Rice, 8. Shell of Malta, 9. Banana peel, 10. Egg shells are in the digestible categories. 1. Polythene, 2. Can, 3. Plastic, 4. Glass, 5. Wire, 6. Gloves, 7. Empty medicine packet, 8. Chips packet, 9. Bottle, 10. Mask. The following devices were used for image capture: Samsung A51 (1080*2400 pixels, 48 MP), HTC 10 (1440*2560 pixels, 12 MP), Redmi Note 9 (1080*2340 pixels, 48 MP), Redmi Note 11 (1080*2400 pixels, 50 MP). The data were cleaned by removing blurry, noisy, and invisible images. Store all data in specific folders based on their categories. Finally, select accurate and clean data for the dataset.
Data source location	This data has been collected from our local places. Such as: • Uttarkhan, Dhaka-1230, Bangladesh. • Abdullahpur, Dhaka-1230, Bangladesh. • Mirpur, Dhaka-1216, Bangladesh. • Azampur, Dhaka -1230, Bangladesh.
Data accessibility	Country: Bangladesh. Repository: Mendeley Data. DOI: 10.17632/96g5pgfnfw.1 URL: https://data.mendeley.com/datasets/96g5pgfnfw/1

## 1 Value of the Data

• The dataset, also referred to as the “BDWaste” dataset, is publicly accessible. The dataset serves as the basis for making well-informed decisions in waste management and allows for assessing the efficiency of waste management strategies.
• The dataset “BDWaste” comprises a comprehensive collection of 2497 images illustrating waste materials, which can be categorized as either biodegradable or non-biodegradable. Thus, researchers can utilize the data to develop an enhanced waste detection model capable of classifying these wastes with greater efficiency.
• Different methods, including machine and deep learning-based approaches, may be used to classify, compare, test, and estimate the data in the dataset.
• The “BDWaste” dataset will help in future research about sustainable environment, non-recycling waste, food waste, and image classification.

## 2 Background

Waste is a wide range of things that people throw away, which causes serious environmental and health problems around the globe. Waste comes from households, industries, animals, minerals, and biomedicine [1,2] and is characterized by its physical state and environmental impact [3]. Bangladesh, with its waste management issues, faces the health risks of untreated waste [4]. Effective waste management strategies are vital for addressing these concerns. Recycling is a crucial component of waste management as it converts rejected materials into valuable resources, thereby promoting economic growth and decreasing overall waste generation [5]. Implementing a proactive strategy involves avoiding environmentally harmful things, such as non-biodegradable polythene bags that cause annual animal fatalities [6]. The research on waste management solutions is crucial for developing new and creative methods to reuse materials and reduce waste to prevent the indiscriminate disposal of waste outdoors, thereby reducing air pollution and minimizing health concerns [7]. In developing countries such as Bangladesh, the efficient handling of waste encounters difficulties as a result of population expansion and the subsequent rise in garbage, leading to significant financial burdens on local governments [8]. Thus, this paper introduces a comprehensive image dataset to support the development of an effective waste management system.

## 3 Data Description

There are many researchers who have worked on waste classification in waste management. In the paper [9], the authors worked on data that contains a total of 25,077 images, with 13,966 organic and 11,111 recyclable. However, in this dataset, data research only worked on the binary classification, more specifically the waste identification. Again, the author of the dataset [10] introduces a waste dataset of 15150 images of different waste. But their collected dataset was only in 12 classes of waste. Also, the author did not consider the biodegradable or non-biodegradable waste.

**Fig. 1:**
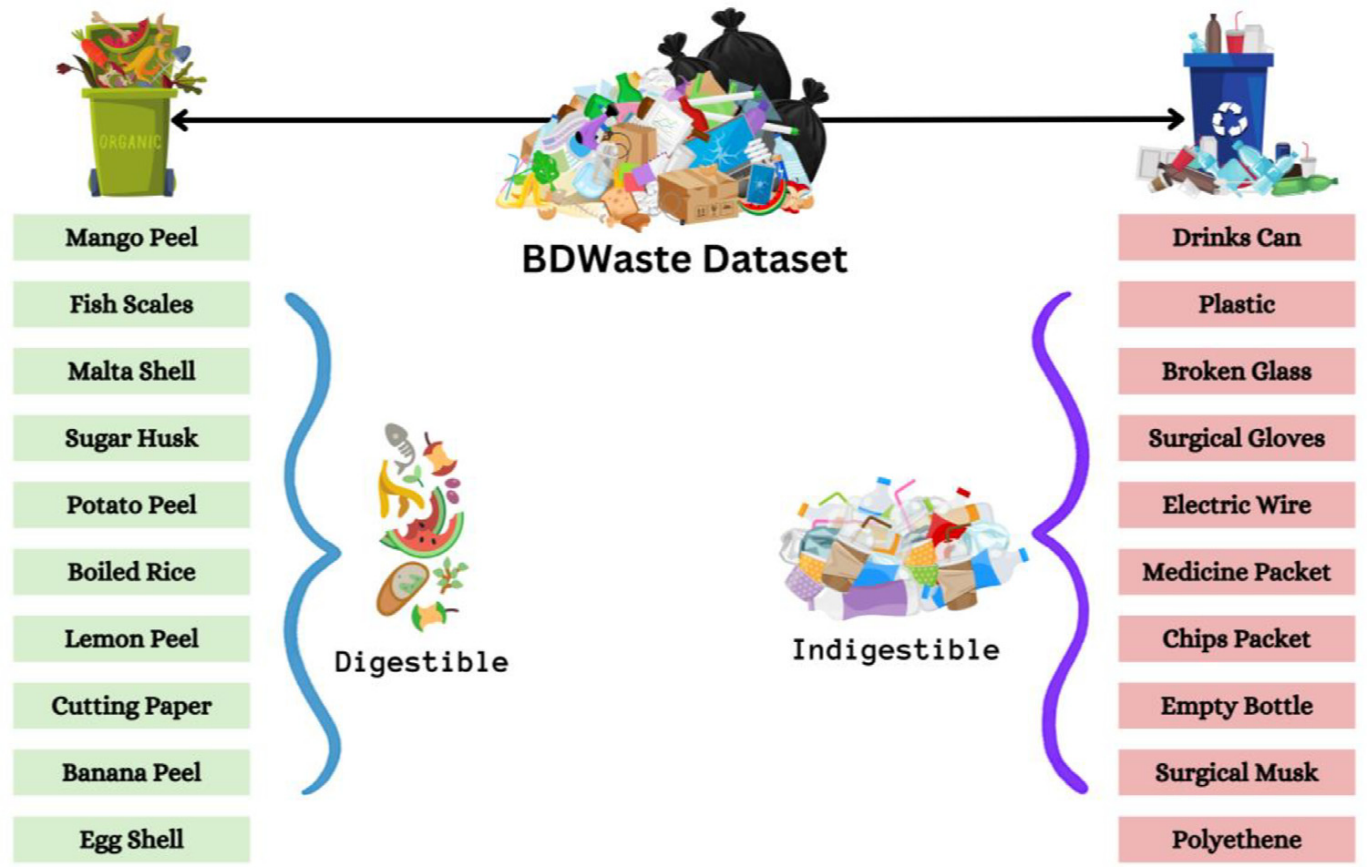
Types of dataset in the “BDWaste” dataset.

**Fig. 2:**
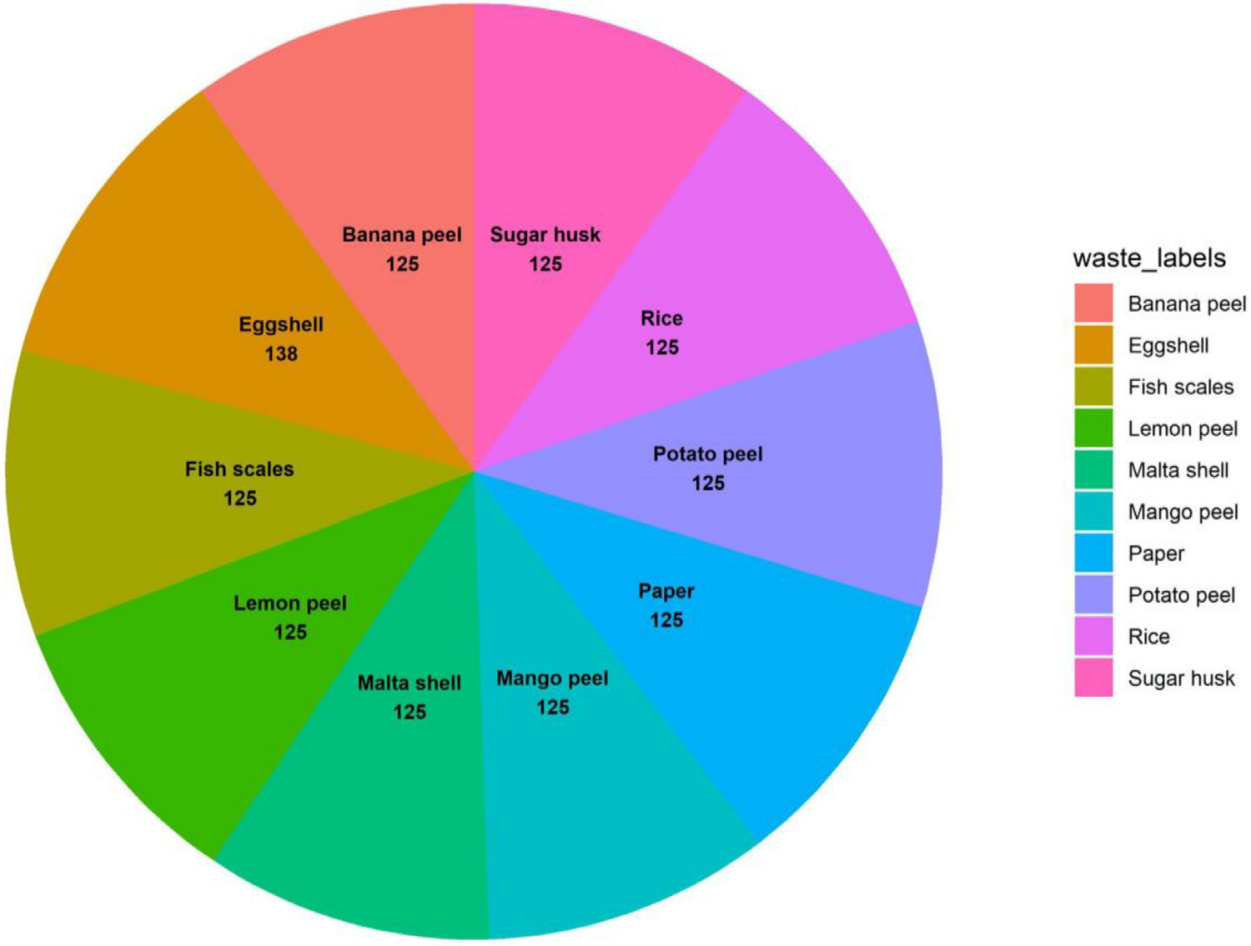
Indigestible categories image distribution in “BDWaste” dataset.

**Fig. 3:**
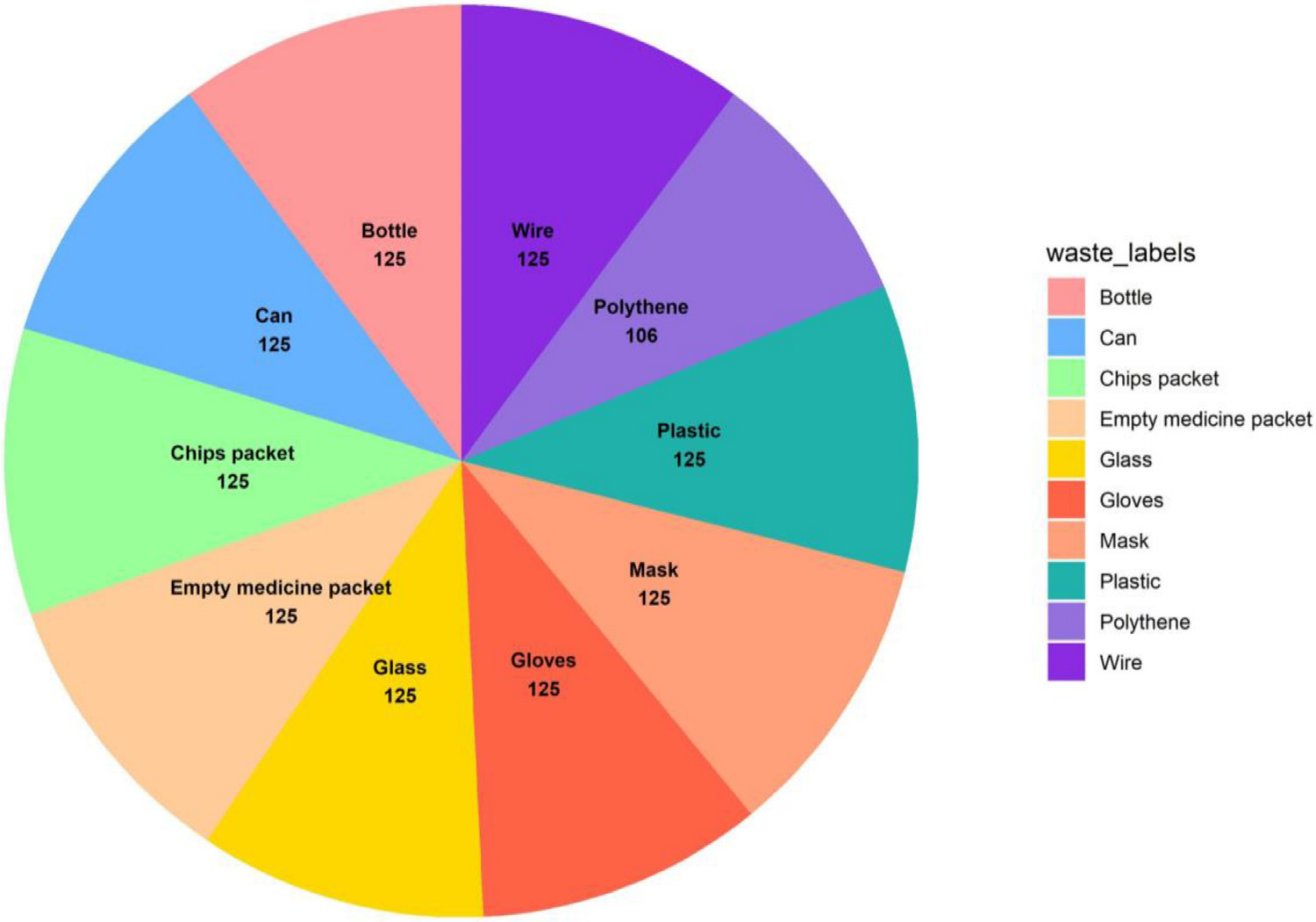
Digestible categories image distribution in “BDWaste” dataset.

Thus, this study presents the “BDWaste” dataset, which is a detailed collection of images that specifically focuses on both biodegradable and non-biodegradable waste in Bangladesh, especially in Dhaka city. The purpose of this dataset is to facilitate the creation of an efficient waste management system, especially in developing countries like Bangladesh that encourages appropriate trash disposal and recycling practices in order to achieve environmental sustainability. The dataset contains a total of 2497 images, of which 1263 belong to biodegradable classes and 1234 belong to non-biodegradable classes. The dataset contains 10 species of digestible and indigestible waste in order to work with a real-time waste management application. Fig. 1 shows the block diagram of the collected types of images in our dataset. The Fig. clearly indicates the collected indigestible waste: a drink can, plastic, broken glasses, surgical gloves, electric wire, and empty medicine packet, chip packet, empty bottle, surgical mask, and polyene. On the other hand, digestible waste includes mango peel, fish scales, Malta shell, sugar husk, potato peel, boiled rice, lemon peel, cutting paper, banana peel, and egg shell. Tables 1 and 2 show a short description of the sample images, along with the number of collected images and their local names. Besides, Figs. 2 and 3 show a pie chart of the distribution of the images in our dataset.

**Table 1:** Data description & quantity of biodegradable waste in the dataset.

Serial	Local name	No. of Raw Images	Description	Sample image
01	Mango peel	125	The mango peel is the primary byproduct of mango. The process of removing and discarding the outer layer of a mango is referred to as mango peeling. Mango peel is highly nutritious, including a variety of nutrients such as vitamins (C and E), antioxidants, and fiber. Upon ripening, the mango skin may maintain its green, yellow, or red hue.	
02	Fish scales	125	Scales are part of the fish. Fish scales are small, rigid plates that cover the skin of most fish. Fish scales protect the fish's skin in many ways. Generally, fish scales are made of hair-like keratin and are found in an overlapping arrangement. We know fish scales are used as waste.	
03	Malta shell	125	The undesirable part that remains after eating Malta is called the Malta shell. Malta is available in all seasons of the whole world. Malta is a good source of vitamin C and is very beneficial for the human body. Malta shell is a biodegradable type of trash. One distinct characteristic of the malta shell is that the outside of the malta shell looks orange.	
04	Sugar husk	125	Sugarcane husk is a fibrous material with cellulose as its main component. Sugar husk is a kind of waste material that comes from sugar. The sugarcane is covered with a hard shell with a soft part inside. All other parts except sugarcane juice are considered waste material. Sugarcane husks easily decompose in soil.	
05	Potato peel	125	Potato peel is the outer layer of the potato that is discarded most of the time before eating. The most common vegetable peel found in the dustbin is potato peel. The potato skin layer is usually thin, and the potato peel is yellowish or white.	
06	Rice	125	Rice is the main source of carbohydrates for the locale. It is widely available in Bangladesh and most parts of the world, but is most commonly consumed in Asia. It has various colors, such as bright white, brown, and red.	
07	Lemon peel	125	Lemon peels are rich in vitamin C, pectin, calcium, potassium, fiber, alpha-hydroxy acids, and flavonoids like D-limousine. From the outside, the lemon peel looks green, light green, or yellow. After extracting the juice of the lemon, in most cases, the pulp is thrown away as waste. It is biodegradable waste.	
08	Paper	125	Paper is a thin, flat material made from crushed wood, bamboo, grass, cottonwood, or cloth. It is used for writing, printing, or drawing. Paper comes in different colors depending on the source of the paper. Paper melts in contact with water, and it is biodegradable.	
09	Banana peel	125	The banana peel is the outer layer of banana. It generally contains fiber, protein, and amino acids. The color of the outer part of the banana peel is yellow (ripe banana) and green (unripe banana). Banana peels are usually 15 to 23 cm long.ssss	
10	Eggshell	138	The outer hard part of the egg is called the eggshell. Its molecular structure mostly consists of calcium carbonate. The eggshell looks white, cream, brown, light green, and bluish-white. Eggshells are also biodegradable.

**Table 2:** Data description & quantity of Non-biodegradable waste in Bangladesh.

Serial	Local name	No. of Raw Images	Description	Sample image
01	Can	125	A can is a container made of aluminum that is silver in color. Cans are used to store soft drinks. It is found as waste in the environment, and it's recyclable waste. A can does not decompose in the soil or in water, so it's harmful to the environment.	
02	Plastic	125	Plastic is a material consisting of a wide range of synthetic or semi-synthetic organic compounds that are malleable and, therefore, can be molded into solid objects. Plastic material is very harmful to the environment. Plastic is used to store water in plastic bottles and other chemicals in chemical laboratories. Plastic melts at high temperatures. Plastic comes in many shapes and colors.	
03	Glass	125	Glass is a solid-like and transparent material that is used in numerous applications in our daily lives. The main component of glass is silica, or silicon dioxide. Glass is solid and doesn't mix with soil.	
04	Gloves	125	Gloves cover the hand with separate sections for the fingers and thumb, sometimes extending over the wrist or part of the arm. Generally, gloves are used to protect hands from colds or various germs. Gloves that are made of nitrile or vinyl-based rubber are not biodegradable. The color of gloves varies, but some common colors are gray, purple, white, blue, and pink. When something pulls on the gloves, they expand and slightly change shape.	
05	Wire	125	A wire is a flexible metallic conductor, typically made of copper. A wire is usually insulated by an outer non-conductive layer and used to carry electric current in a circuit. There are different types of wires, and they can consist of various components such as plastic, copper, and elastic. Common colors of wires are white, black, blue, red, and green. Wire does not mix with the soil.	
06	Empty medicine packet	125	Packets used for medicine preservation are medicine packets. Plastic is the raw material for medicine packets. The top is coated with aluminum to prevent air from entering. An empty medicine packet doesn't mix with the soul.	
07	Chips packet	125	A chip packet used for storing chips is a packet of chips. It is made of plastic, or polythene. Polythene, or plastic, doesn't mix with the soil.	
08	Bottle	125	A bottle is a container made of an impermeable material. (such as plastic, glass, or aluminum), it has various shapes and sizes that store and transport liquids. When the bottle expires after a certain period of time, it is thrown away as waste.	
09	Mask	125	A mask is a product that covers the wearer's nose and mouth. The mask protects from various germs and dust. It is usually made of cotton, rubber, nylon, and cloth; hence, it is not biodegradable.	
10	Polythene	106	Polythene is a thermoplastic polymer. It is one of the most widely used plastics in the world; every year, 10 million tons of polyethylene are produced worldwide. Polythene is a harmful material for the environment.

## 4 Experimental Design, Materials and Methods

**Fig. 4:**
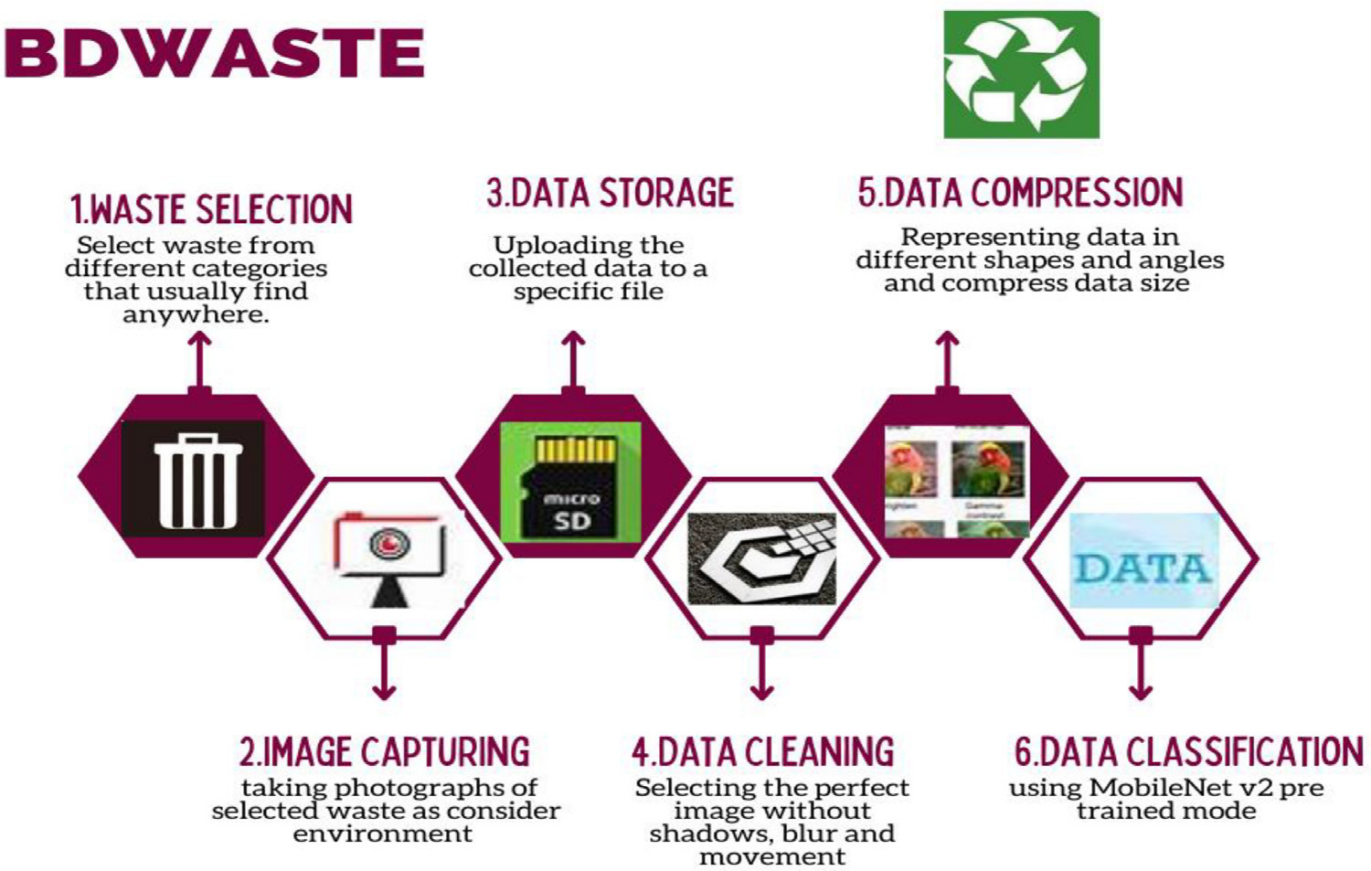
Graphical abstract of the “BDWaste” dataset building procedure [11].

The research follows several steps to collect the data from the different regions in Bangladesh. The Fig. 4 shows the graphical abstract of the research [11], [12], [13].

In this figure, a set of interconnected the steps has been interpreted whose help us to build the dataset. The subsequent procedure includes the waste selection, image capturing, data storing, image cleaning, data compression and finally the application of the Deep Learning (DL) to classify the digestible and indigestible waste.

### 4.1 Waste selection

The research mainly focuses on the two type of types waste such as biodegradable and non-biodegradable. The identification of the waste are given as follows:

**Biodegradable** refers to all waste that is mixed with soil. These wastes are not harmful to the environment, but they sometimes pollute the air. However, these wastes can be converted into fertilizer through proper processing or management. From the biodegradable categories, we have selected 11 distinct species. The species are:

• **Potato peel**: The potato skin layer is usually thin. Rough brown or light brown in appearance. Black spots are also seen on the skin. Potato peel is yellowish-white or white inside.
• **Lemon peel**: The skin layer of the lemon is usually of medium thickness. From the outside, the lemon peel looks green, light green, or yellow. Also, green and yellow are mixed colors too. Almost white from the inside. Lemon peel also has another layer between its inner and outer coverings.
• **Rice**: Rice looks bright white, brown, or red in color (but waste is usually yellow, so rice is discarded). Rice is soft and small in size.
• **Sugarcane husk**: The outer part of the sugarcane husk is green, light green, brown, or red in color, which is hard. The inner part of the sugarcane husk is gray-white, greenish-white, and softer than the outer part. Sugarcane husks are of any size, big, small, or medium in length.
• **Fish scale**: The scale of a fish consists of three characteristic layers. Fish scales look like a small, rigid plate. The color of the fish scale usually has a silvery appearance. Also, its pigment types range from dark to light: black, gray, brown, orange, yellow, and red.
• **Malta Shell**: Some distinguishing characteristics of malta shells are: From the outside, the malta shell looks orange or green in color. Malta shell attached. It has a thin coating between its inner and outer layers, which is white or yellowish white. From the inside, the malta shell looks orange-white in color.
• **Paper**: The paper looks like a thin sheet. Paper is of different colors (white paper is mostly used). Paper melts in contact with water.
• **Banana peel**: Some features to identify banana scabs: The outer covering of the banana peel is yellow (ripe banana), green (unripe banana), and yellow-green mixed colors. The peel turns black after being removed from the banana. The inner layer of banana peel looks white—yellowish white. Banana peels are usually 15 to 23 cm in size.
• **Egg Shell**: The outer hard shell of the egg (egg shell) looks white, cream, brown, light green, or bluish white. A very thin white coating is present as a layer between the egg and the eggshell. The shape of the eggshell can vary depending on how the shell is broken.
• **Coffee Cup**: Coffee cups can be of different colors and designs, but most coffee cups are white. The coffee cup is not too big—about 8 to 10 oz. The front of the coffee cup is slightly rounded at the beginning.
• **Mango peel**: Mango peel looks yellow, red, orange (ripe mango), green, and light green (raw mango) from above. The inner part of mango peel is dark yellow, yellow, or light yellow. Mango peel dries up after 2 or 3 days and becomes very dark in color. Depending on the size of the mango, the peel is of different sizes.

**Non-biodegradable** refers to waste that does not mix with soil. Moreover, these wastes are harmful to the environment. Also, some of these species form a layer in the soil that prevents plants or water from penetrating through this layer to reach the soil. Choose 11 different species from the non-biodegradable category. The species are

• **Polythene**: Polyethylene has a thin, light structure. Generally, polythene is seen in white, blue, green, red, black, etc. colors.
• **Can**: The can is made of aluminum, which is bright silver in color. The logos of different brands are placed on the can to show different colors and sizes. Sunlight is reflected in the can.
• **Glass**: Glass is a transparent, shiny metal. Glass has the ability to conduct both hot and cold temperatures. Glass is a hard metal.
• **Plastic**: Plastic is a somewhat hard metal. Plastic melts at high temperatures. Plastic comes in many shapes and colors.
• **Wire**: Depending on the connection, the wire can be white, black, blue, red, or green. The wire usually has two to three coats. The inner casing is made of copper.
• **Gloves**: Gloves are shaped like hands. White, blue, or yellowish white (may vary). The gloves get bigger when pulled. Resilient religion prevails.
• **Chips Packet**: A packet of chips looks different from the outside but is bright silver inside. About 7.5 to 9.5 inches in height and 5 to 6 inches in width.
• **Empty Medicine Packet**: The upper part is covered with plastic, which is transparent or silver in color. A bright coating of aluminum is provided to carry the drug information. Usually, 10 furrows are cut.
• **Mask**: The mask is 5 to 7 inches. The mask looks light blue, white, or black from the outside. The inside of the mask is white. Two ends have two brackets.
• **Bottle**: Bottles range in size from 250 ml. White and green in color. The upper part is narrow, and the lower part is spread.

### 4.2 Image capturing

**Fig. 5:**
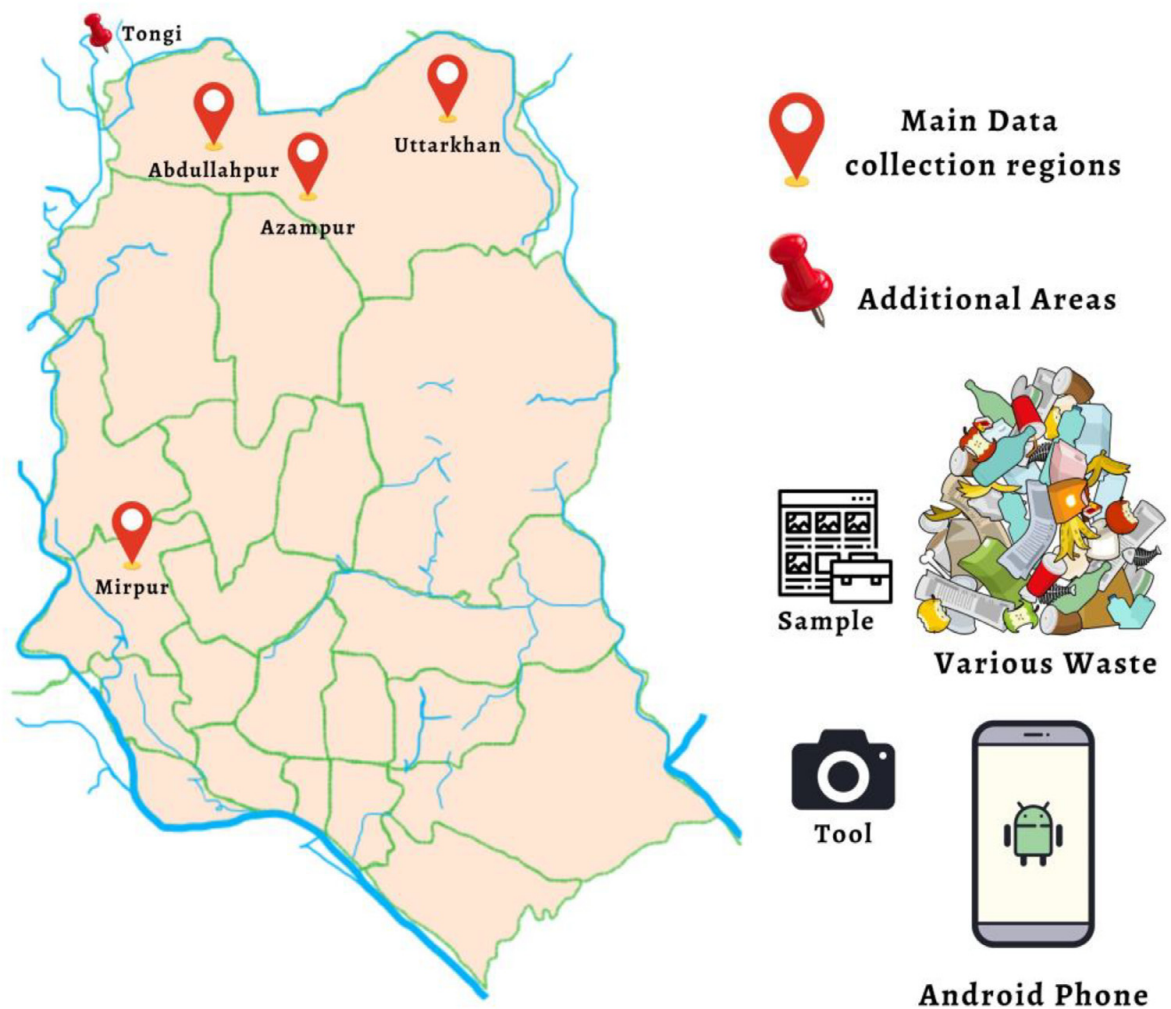
Map of the Dhaka city, Bangladesh and the waste image collected regions.

Raw data from the “BDWaste” dataset was collected from various roadsides and local dustbins. Fig. 5 shows the map of Dhaka City, Bangladesh, and the locations where we have collected the waste. The Android devices were used to collect waste data from different regions of Dhaka city. Table 3 shows the configuration details of the devices employed during the data collection. We have considered that a daylight environment was set to avoid image shadows during data collection. To improve data quality and reduce shadows, we employed diffuse lighting.

**Table 3:** Device name and configuration details.

Device name	Camera Resolution
Samsung A51	1080*2400 pixels, 48 MP, f/2.0, 26mm,1/2.0", 0.8µm, PDAF12 MP, f/2.2, 123˚, 5 MP, f/2.4, 5 MP, f/2.2,)
HTC 10	1440*2560 pixel 12 Mp,f/1.8-5MP,f/1.8
Redmi Note 9	1080*2340 pixels,48 MP, f/1.8, 26mm,1/2.0", 0.8µm, PDAF 8 MP, f/2.2, 118˚,1/4.0", 1.12µm 2 MP, f/2.4, AF 2 MP, f/2.4.
Redmi Note 11	1080*2340-pixel,50 MP, f/1.8, 26mm, 1/2.76", 0.64µm, PDAF 8 MP, f/2.2, 118˚, 1/4", 1.12µm 2 MP, f/2.4, 2 MP, f/2.4.

During the image capture process, images were taken from different angles, sides, and low-lights to fully understand the variation and characteristics of the waste. Blurred, overlit, and moving images are removed after image acquisition, which is a laborious process in data collection. By eliminating these inferior images, we ensured the integrity of the dataset and improved its reliability [12].

**Fig. 6:**
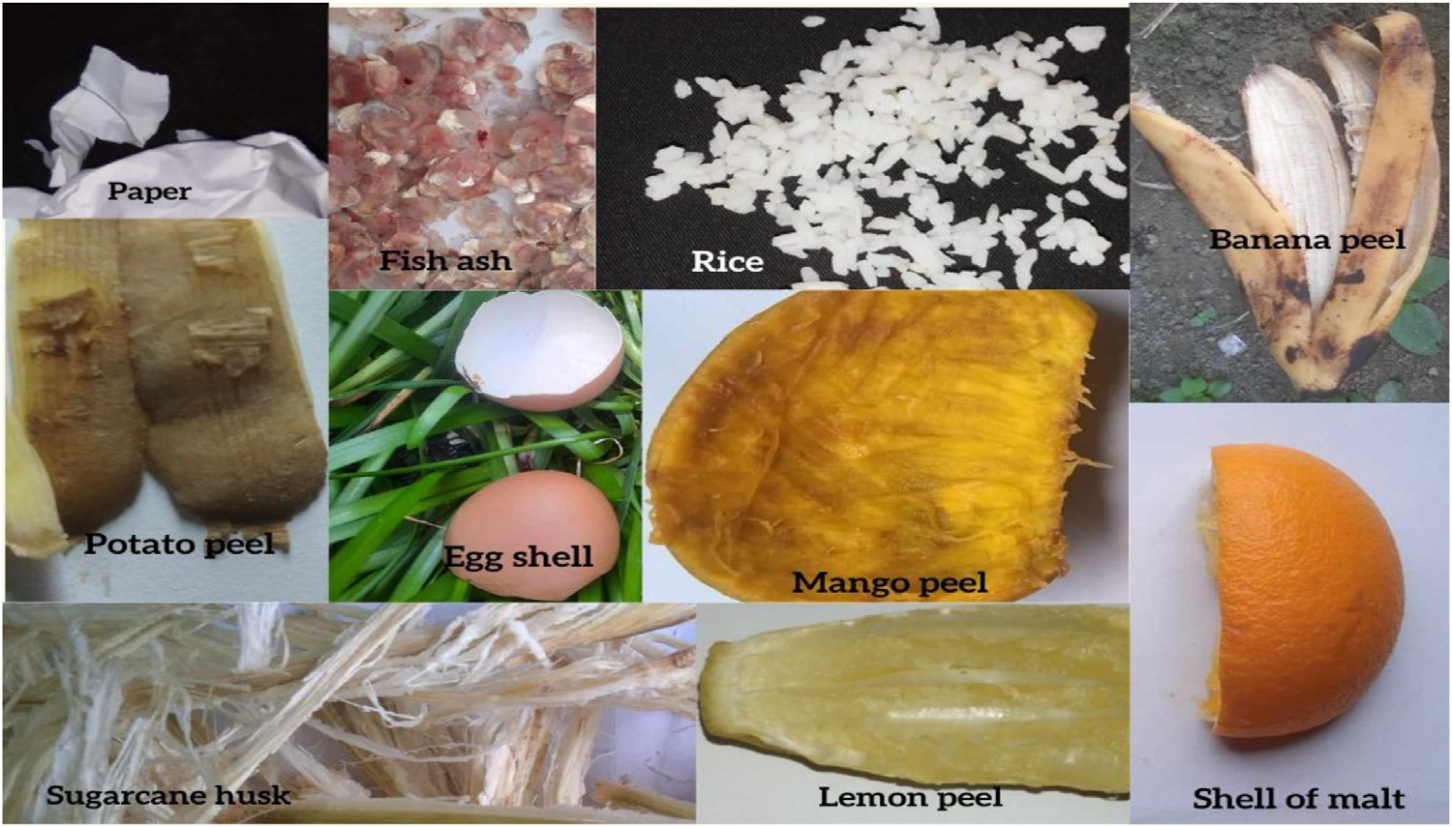
Sample images of the biodegradable waste.

**Fig. 7:**
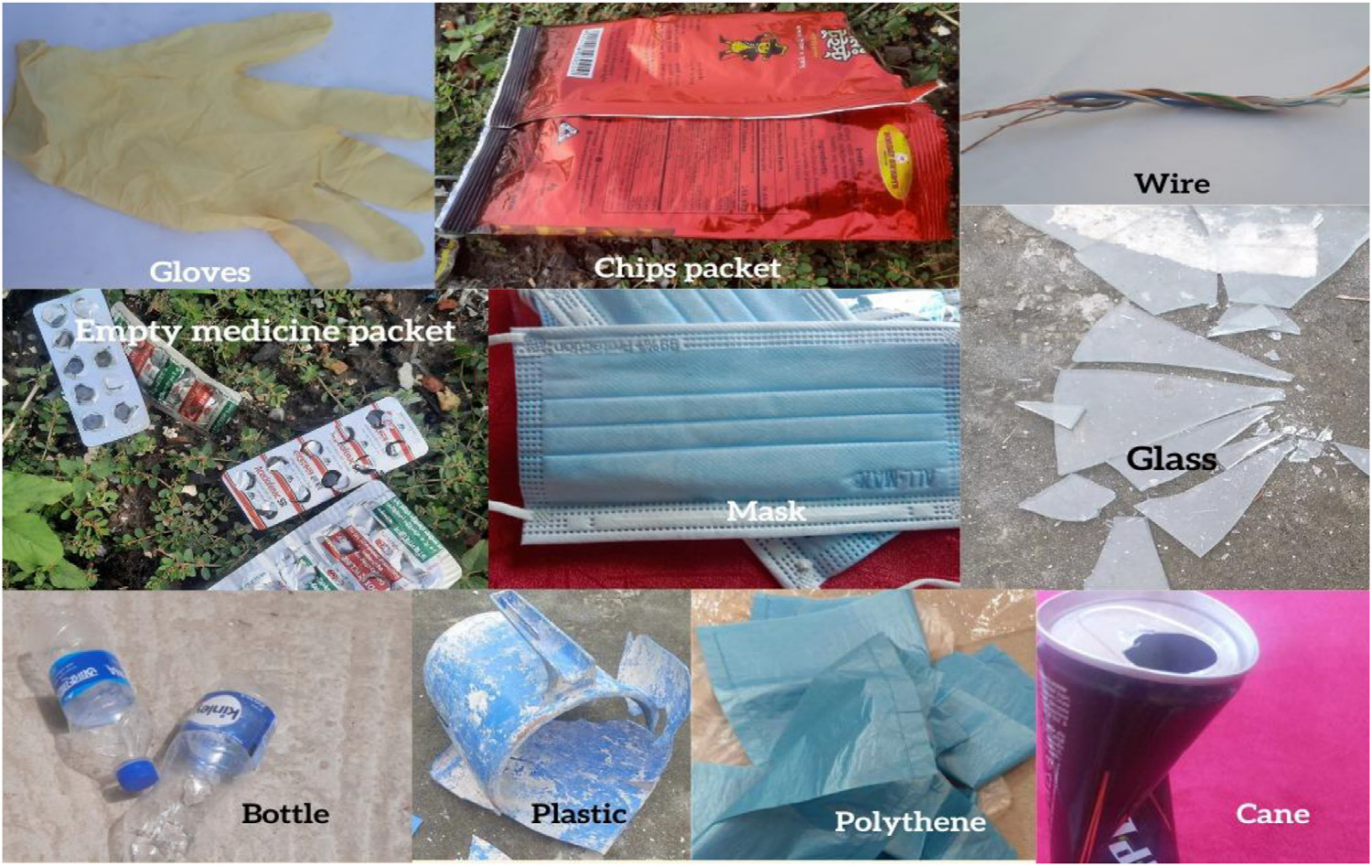
Sample images of the non-biodegradable waste.

On the other hand we have provided the samples from the “BDWaste” dataset. Figs. 6 and 7 shows the sample captured images according to the types.

### 4.3 Image storing and folder management

After collecting the data, store it somewhere for later use. In a word, saving the data to a specific drive. Each piece of data in the dataset is kept in a separate folder according to the data classes. We have created two separate folders where one folder renamed as ‘digestible’ and another folder rename with “Indigestible”. Each folder contains the images according to the local name. We have also rename the each individual images in numeric form.

### 4.4 Image cleaning (data cleaning)

Images with low resolution, insufficient lighting, obscured details, and motion blur are eliminated from the data storage. Additionally, verify the dimensions and file type of each image and store all the information. Initially, a total of more than 2600 images were collected from various locations. The images vary in size and were captured using several models of smartphones. During the collection of images, those that showed poor quality, blurriness, overexposure, noise, or motion were subsequently deleted. The dataset has an overall number of 2497 original images after performing image processing. In the process of data cleaning, the objective is to create impeccable data collection in order to achieve a clean dataset.

### 4.5 Image compression

Image compression involves compressing the size of the folder. In this research, the image compression technique involves a lossless compression technique [14]. Our prime objective was to preserve the quality of the images while reducing the size of the master folder. We have written the Python code to preserve the image quality and the size of the images. Before the image compression, the data folder was 10.02GB. But, after applying the compression technique, we have found a total size of 6.65GB.

### 4.6 Waste classification using deep learning

The research also performs Deep Learning (DL) on the collected images in order to check the integrity of the dataset and whether the collected images are compatible for future applications. To apply DL, we have applied two pre-trained Convolutional Neural Network (CNN) models, such as InceptionV3 and MobileNetV2. We have trained these models in Google Colab which has a dedicated Graphics Processing Unit (GPU) and 53GB of RAM.

**Fig. 8:**
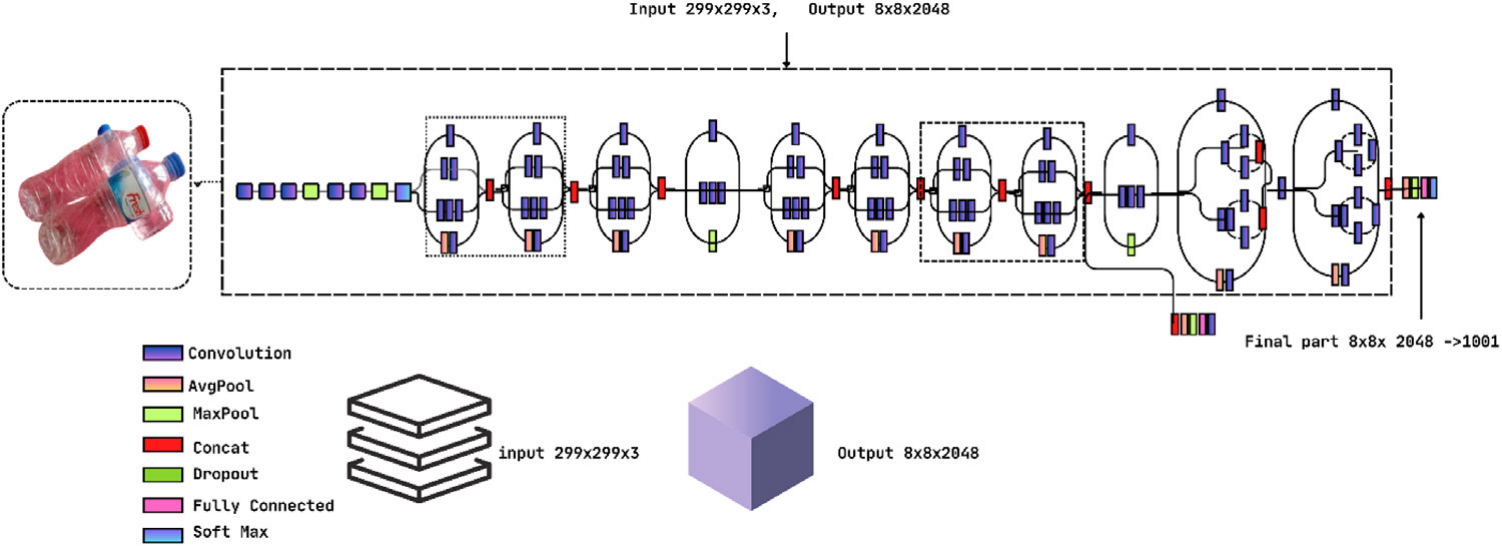
Architecture of the InceptionV3 model.

**Fig. 9:**
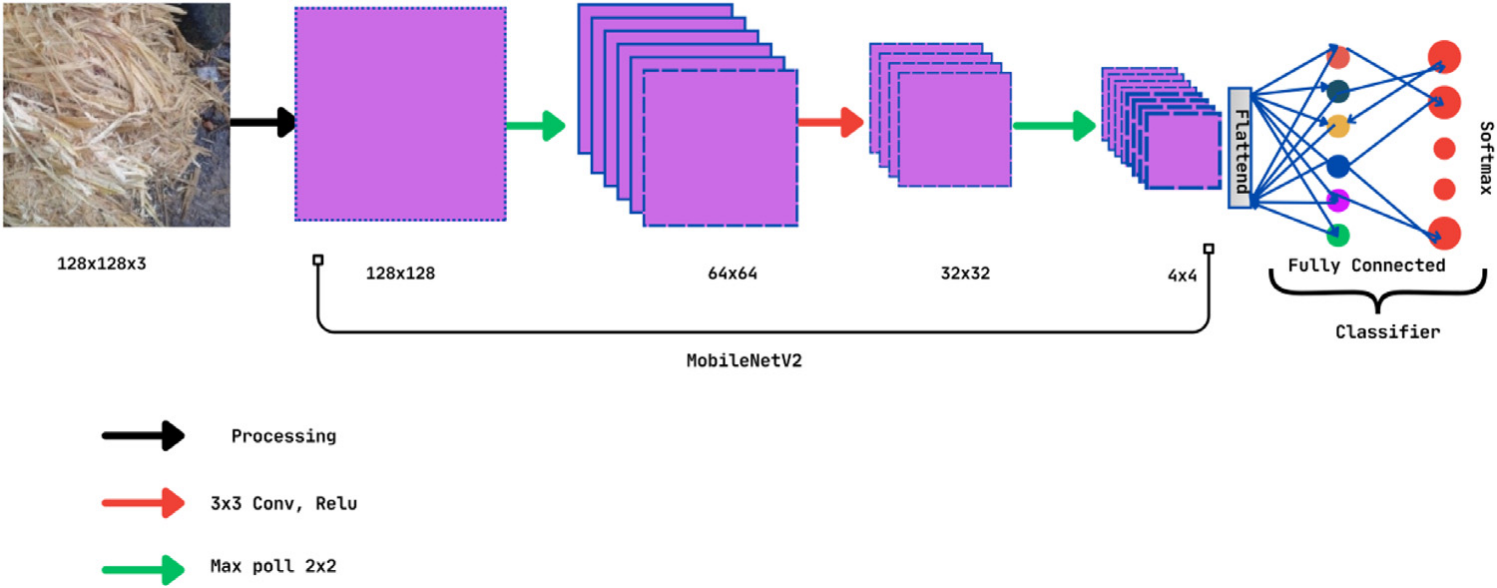
Architecture of the MobileNetV2.

Figs. 8 and 9 show the architecture of the InceptionV3 and MobileNetV2 pre-trained CNN models [15,16]. This Fig. clearly shows the architectures of these two models with input-output layers and convolutional layers. We have considered a set of parameters to train the models and determine their accuracy. Table 4 shows the parameters for both models in the training and testing of the dataset. The research adopted “Adam” as an optimized version of both pre-trained models. To make a consistent model, we have utilized 20 epochs for both of these two models and the same batch size of 32.

**Table 4:** Summary of the pre-trained CNN models.

Model Name	Epochs	Optimizer	Training Parameter	Non-Trainable Parameter	Batch Size
InceptionV3	20	Adam	20,490	21,802,784	32
MobileNetV2	20	Adam	12,810	2,257,984	32

We have applied the training of the models with a train and test ratio of 80% and 20%, respectively. To find the efficacy of the models, we have enumerated the performance of these models with evaluation matrices in order to check the integrity of the dataset in DL applications. Table 5 shows the summary of the performance evaluation matrices that were considered for this study. Based on the evaluation matrices, we have interpreted the experimental results. Table 6 illustrates the experimental results from the two models in waste classification. In this, we have segmented the training into two steps. Firstly, we have applied the indigestible waste classification. In this step, we have applied the InceptionV3 and MobileNetV2 models. After that, we applied these two pre-trained CNN models to digestible waste. We have found the highest accuracy of 96.87% with the MobileNetV2 models in the indigestible waste classification and 99.70% with the MobileNetV2 models in the digestible waste classification.

**Table 5:** Description of performance evaluation matrices.

Metrics	Description
Accuracy (%)	Displays the overall right prediction percentage.
	$A=\frac{TP+TN}{FP+TP+FN+TN}\times 100$
Precision (%)	Describes as a way to assess a model's quality.
	$P=\frac{TP}{TP+FP}\times 100$
Recall (%)	Defines as a measurement of model quantity.
	$R=\frac{TP}{TP+FN}\times 100$
F1-score (%)	Demonstrates how reliable and accurate a model is.
	$F1=2\times \frac{R\times P}{R+P}\times 100$

**Table 6:** Performance of the pre-trained CNN models in waste classification on “BDwaste” dataset.

Waste Types	Pre-trained Model Name	Accuracy (%)	Precision (%)	Recall (%)	F1-score (%)
Indigestible	InceptionV3	96.87	97.50	96.87	96.92
	MobileNetV2	96.87	97.65	96.87	96.90
Digestible	InceptionV3	98.02	98.13	97.97	98.15
	MobileNetV2	99.70	99.72	99.69	99.71

**Fig. 10:**
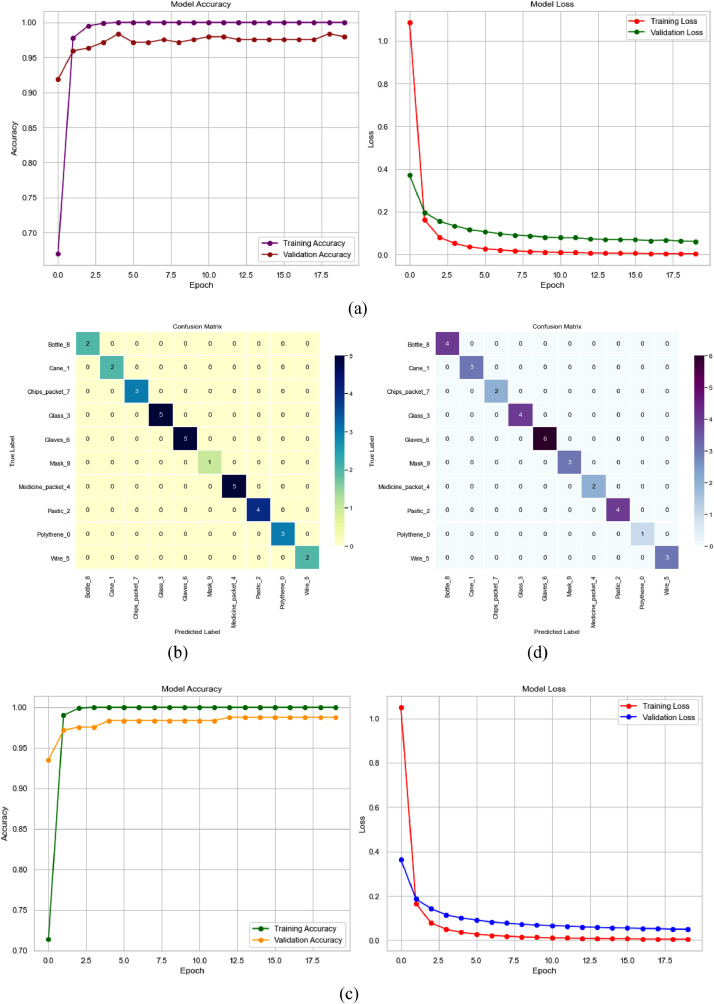
Performance measurement from InceptionV3 with (a) Learning Curve (b) Confusion Matrix and from MobileNetV2 with (c) Learning Curve (b) Confusion Matrix in indigestible waste classification.

**Fig. 11:**
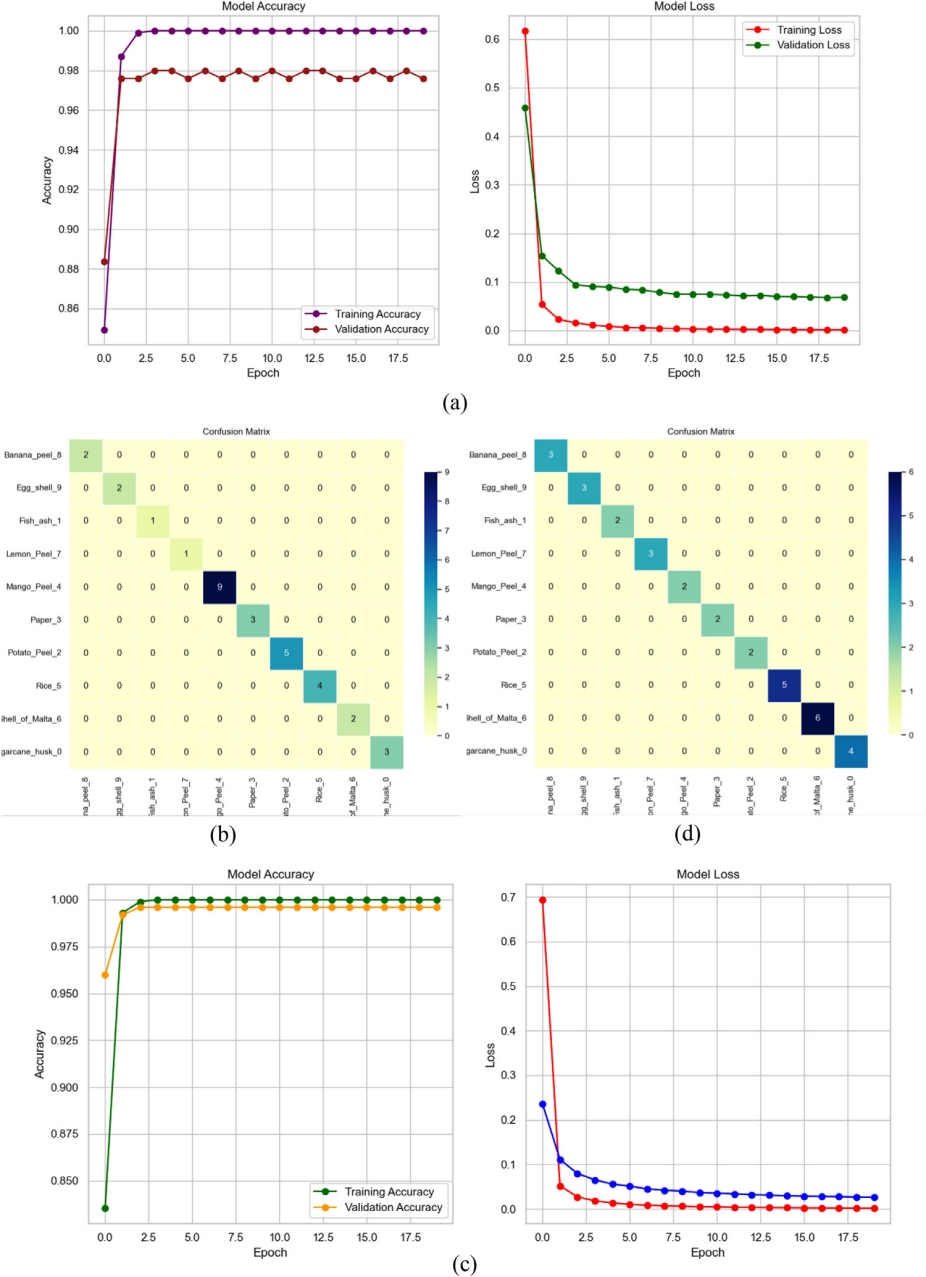
Performance measurement from InceptionV3 with (a) Learning Curve (b) Confusion Matrix and from MobileNetV2 with (c) Learning Curve (b) Confusion Matrix in digestible waste classification.

Further, we have provided the learning curves after 20 epochs as well as the confusion matrix for each class of waste. Fig. 10 shows the summary of the performance in indigestible waste classification with learning curve and confusion matrix produced from the InceptionV3 and MobileNetV2 model. On the other hand, Fig. 11 shows the performance of digestible waste classification with respective graphs.

## Limitations

While working with the proposed model and the collected “BDWaste” dataset, the research found some drawbacks in both data collection and Deep Learning (DL) model training. Firstly, Bangladesh is dealing with a huge amount of waste, but our attention was specifically on the Dhaka region, where we chose to study only 20 specific types of waste. Secondly, there are many different kinds of waste in Dhaka and throughout Bangladesh, like empty medicine packets. But our focus was limited to just a few specific types of empty medicine packets. Thirdly, it's important to note that waste in other parts of the world can vary significantly. However, our research only focuses on Bangladeshi waste in two significant types: biodegradable and non-biodegradable. Fourthly, the research only used DL in order to check and assess the performance of the computer vision models in waste classification. Also, the research only applied two pre-trained CNN models to the waste classification. In the future, we are determined to find solutions to overcome these challenges. A key part of our strategy involves creating a smart bin, essentially a robotic waste bin, and we've chosen the Bengali name “***Moylar Jhuri***” for it. The smart bin will be embodied with a set of Internet of Things (IoT) sensors, a microprocessor such as a Raspberry Pi, and a camera module to capture real-time images of the waste. We have a plan to apply the YoloV5 model to real-time waste detection and build up the model on the Raspberry Pi in order to cope with biodegradable and non-biodegradable waste. Additionally, we aim to work with blurry, moving, or unclear images to check and test how well our proposed models perform. Furthermore, we'll evaluate the performance of various DL models based on their time and space complexity.

## Ethics Statement

The ethical concerns pertaining to the dataset on Waste involve a dedication to conscientious data collecting and usage. The production of this collection is guided by principles that prioritize justice, openness, and environmental respect. Considerable effort was made to ensure that the collecting procedure was conducted in a manner that did not cause harm to any live animals or ecosystems. It is imperative to acknowledge that all waste images were obtained with explicit consent from owners. The authors have thoroughly reviewed and adhered to the ethical guidelines for publishing in Data in Brief. They have ensured that their research does not involve human subjects, animal experimentation, or the use of data obtained from social media platforms.

## CRediT authorship contribution statement

**Wahidur Rahman:** Data curation, Formal analysis, Project administration, Supervision, Writing – review & editing. **Mohona Akter:** Data curation, Formal analysis, Software, Conceptualization, Methodology, Investigation, Writing – original draft, Visualization. **Nahida Sultana:** Data curation, Formal analysis, Writing – original draft, Resources, Conceptualization, Methodology, Software, Investigation. **Maisha Farjana:** Data curation, Formal analysis, Project administration, Supervision, Writing – review & editing, Methodology, Resources. **Arfan Uddin:** Data curation, Writing – review & editing. **Md. Bakhtiar Mazrur:** Writing – review & editing. **Mohammad Motiur Rahman:** Formal analysis, Conceptualization, Project administration, Supervision, Writing – review & editing.

## Data Availability

BDWaste: A comprehensive image dataset digestible and indigestible waste in Bangladesh (Original data) (Mendeley Data).
